# Utility of the Intelligibility in Context Scale for Predicting Speech Intelligibility of Children with Cerebral Palsy

**DOI:** 10.3390/brainsci11111540

**Published:** 2021-11-20

**Authors:** Jennifer U. Soriano, Abby Olivieri, Katherine C. Hustad

**Affiliations:** 1 Waisman Center, University of Wisconsin–Madison, Madison, WI 53705, USA; kchustad@wisc.edu; 2 Department of Communication Sciences & Disorders, University of Wisconsin–Madison, Madison, WI 53706, USA; aolivieri2@wisc.edu

**Keywords:** cerebral palsy, Intelligibility in Context Scale, transcription speech intelligibility

## Abstract

The Intelligibility in Context Scale (ICS) is a widely used, efficient tool for describing a child’s speech intelligibility. Few studies have explored the relationship between ICS scores and transcription intelligibility scores, which are the gold standard for clinical measurement. This study examined how well ICS composite scores predicted transcription intelligibility scores among children with cerebral palsy (CP), how well individual questions from the ICS differentially predicted transcription intelligibility scores, and how well the ICS composite scores differentiated between children with and without speech motor impairment. Parents of 48 children with CP, who were approximately 13 years of age, completed the ICS. Ninety-six adult naïve listeners provided orthographic transcriptions of children’s speech. Transcription intelligibility scores were regressed on ICS composite scores and individual item scores. Dysarthria status was regressed on ICS composite scores. Results indicated that ICS composite scores were moderately strong predictors of transcription intelligibility scores. One individual ICS item differentially predicted transcription intelligibility scores, and dysarthria severity influenced how well ICS composite scores differentiated between children with and without speech motor impairment. Findings suggest that the ICS has potential clinical utility for children with CP, especially when used with other objective measures of speech intelligibility.

## 1. Introduction

Speech intelligibility is a critical clinical consideration for children with cerebral palsy (CP). Studies suggest that about half of children with CP may have dysarthria [1,2,3], a speech motor disorder that impacts one or more of the speech subsystems (i.e., articulation, phonation, resonance, respiration for speech, and prosody), frequently resulting in reduced intelligibility. Recent work indicates that even children with CP who do not have clinical dysarthria may lag behind their peers in speech intelligibility development [4,5,6]. Enhancing speech intelligibility is a key objective of treatment for individuals with CP and dysarthria [7]. Consequently, the measurement of intelligibility and our ability to consider it in a developmental context is of the utmost importance.

Until very recently, comprehensive normative standards for characterizing intelligibility development in typical children did not exist. The most recent work, however, has provided a foundation for understanding growth trajectories between the ages of 2 and 10 years [8,9,10]. Results have indicated that children’s intelligibility grows rapidly in the preschool years and is highly variable. Intelligibility continues to improve with age throughout middle childhood, and variability decreases with age [8,9,10]. Studies of typical speech intelligibility development provide an important foundation for interpreting intelligibility in children with dysarthria. Hustad and colleagues investigated single-word [11,12] and multi-word [13] intelligibility development in children with CP longitudinally from 2–8 years of age. Results showed that, similarly to their typically developing peers, children with CP continued to develop intelligibility through the school age years. However, they tended to achieve intelligibility milestones later than their typically developing peers. One clear finding was that the presence of dysarthria had an important impact on intelligibility outcomes. For example, the period of steepest growth for multi-word intelligibility occurred more than a year later for children with CP and dysarthria than for children with CP who did not have dysarthria. In addition, all children with CP, regardless of dysarthria status, had their steepest growth later than typical peers.

The aforementioned studies on the development of intelligibility in typically developing children and children with CP used a highly structured and controlled measurement paradigm, following the standard of practice in motor speech disorders research and clinical practice [14,15,16,17]. However, this type of environment is different than real world communication contexts and most closely reflects a measure of “activities” in the World Health Organization’s International Classification of Functioning, Disability and Health (ICF) framework [18].

In an attempt to capture a more wholistic impression of intelligibility, Sakash and colleagues [19] asked parents of children with CP to make subjective ratings of how understandable they felt their child was to others. They then examined the relationship of parent ratings of intelligibility to multiword transcription intelligibility scores. Results indicated that parent ratings of children who had intelligibility between 90% and 99% were less varied and more comparable to their child’s transcription intelligibility scores than parent ratings for children who had intelligibility lower than 90%. Subjective ratings for children with intelligibility below 90% showed considerable variability. This variability was especially noteworthy for children in the middle of the severity continuum. Findings suggested that subjective parent ratings of intelligibility may not be valid representations of objective clinical measures of speech intelligibility. The authors noted that the Intelligibility in Context Scale (ICS) [20], which queries parent perceptions of a child’s intelligibility with different communicative partners, may better capture the relationship between parent ratings of intelligibility and objective measures of speech intelligibility (i.e., transcription intelligibility scores).

The ICS [20] has received widespread attention for its characterization of children’s intelligibility. The ICS is a questionnaire that aims to measure parental perception of their child’s speech intelligibility with familiar and unfamiliar partners in day-to-day communication contexts. Thus, it aims to capture “activities” and “participation” level functioning within the ICF framework [21]. The ICS has seven questions that ask parents to rate how well different communication partners understand their child’s speech. Communication partners include parents themselves, immediate family, extended family, friends, acquaintances, teachers, and strangers. Parents make ratings using a 5-point ordinal scale (i.e., 5—Always, 4—Usually, 3—Sometimes, 2—Rarely, and 1—Never). An advantage of such rating scales is ease of administration, which is valuable in a clinical setting [22].

Mcleod and colleagues conducted a normative validation study of the utility of ICS among English speaking children [21]. Their results showed the clinical utility of the ICS as a screening tool of preschool children’s speech intelligibility. The ICS has also been used as a measure of speech intelligibility in children and adults in various research studies [20]. For example, Veenhuis and collaborators [23] used the ICS to quantify speech intelligibility of children and adults with ataxia telangiectasia and dysarthria. Studies examining the impact of partner familiarity on parents’ perception of their child’s intelligibility have revealed that ICS scores were on average highest with parents and lowest with strangers [24,25,26,27,28].

The ICS has been translated into more than 60 different languages (accessible from https://www.csu.edu.au/research/multilingual-speech/ics; accessed on 14 October 2021) [29]. As part of the validation of several of the translated versions, the relationship of the ICS composite score and articulatory measures of speech (e.g., percent of consonant correct) has been examined. Across studies, results have shown correlations of varying strength between ICS composite scores and articulatory measures of speech (ranging from no correlation to moderate correlation) [24,25,26,27,28]. Lagerberg and colleagues suggested that the ICS, intended to be a measure of intelligibility, may be best compared to other measures of speech intelligibility, such as transcription intelligibility scores [30]. However, only two studies have examined the relationship between ICS scores and transcription intelligibility scores. These studies examined children with speech sound disorders (SSD) [30] and children with velopharyngeal insufficiency (VPI) [31]. Hosseinabad and associates noted a strong significant correlation between ICS scores and transcription intelligibility scores of children with VPI, indicating the potential clinical utility of the ICS as a proxy for objective measures. However, Lagerberg and colleagues reported no statistically significant correlation between ICS scores and transcription intelligibility scores in children with SSD. Discrepant findings on the relationship between ICS scores and transcription intelligibility scores highlight the need for further investigation.

In the current study, we sought to quantify the relationship of ICS scores to transcription measures of speech intelligibility for children with CP. Our primary interest was to examine the clinical utility of the ICS as a proxy for transcription intelligibility when assessing children with CP. This study addressed the following specific research questions:

How well do ICS composite scores predict intelligibility scores as measured by orthographic transcription by unfamiliar listeners?

Do individual questions from the ICS differentially predict transcription intelligibility scores?

How well do ICS composite scores differentiate between children with and without speech motor impairment?

Given the varied results from the current literature, and in particular the work of Sakash and colleagues [19], we expected that ICS composite scores would be most strongly related to intelligibility for children at the extreme ends of the severity continuum (i.e., most severe and least severe), and that the children in the middle of the continuum would show varied results. Collectively, we expected that overall ICS scores would be a moderately strong predictor of intelligibility scores. We expected to see differential effects for the various individual item questions (i.e., communication partners), however, we did not have hypotheses regarding which items would be the strongest predictors. The ICS has been recommended as a screening tool in children with SSD and a clinical tool for measuring functional intelligibility in children with VPI based on previous studies. Findings suggest the potential capacity of the ICS to differentiate children with and without speech concerns. Therefore, for question 3, we expected that ICS composite scores would show meaningful differentiation between children with CP who have speech motor impairment (dysarthria) and those with CP who do not have speech motor impairment (no dysarthria), to the extent that speech motor impairment results in reduced intelligibility.

## 2. Materials and Methods

### 2.1. Participants

Ethical approval for this research was granted by the University of Wisconsin-Madison Institutional Review Board (Social and Behavioral Sciences). All participants in the study provided voluntary consent to participate.

#### 2.1.1. Children with Cerebral Palsy

Children in current study were part of a longitudinal project on the development of communication skills in children with CP. Eligibility criteria for the larger study required participants to have a medical diagnosis of CP and normal hearing. Participants were recruited through medical clinics in the upper Midwest region of the USA. As part of the longitudinal study, each participant completed a standard data collection session that spanned up to 3 h in length. Following a research protocol, a speech-language pathologist (SLP) assessed speech, language, cognitive, oral-motor, hearing, and communication abilities in a sound attenuating suite. Parents completed questionnaires prior to their child’s session. Participants were included in the current study if they completed the Test of Children’s Speech (TOCS+) [32], and their parents completed the Intelligibility in Context Scale (ICS) [21] for the same visit. Forty-eight participants, comprising 28 boys and 20 girls, met the inclusion criteria. Mean age was 12.82 years (SD = 1.94).

As part of the assessment session and following a well-established protocol [33], children were classified as to the presence or absence of speech motor impairment by two certified SLPs with expertise in CP; classification agreement was 96%. Classification was based on perceptual assessment of each child’s speech, presence of drooling and/or facial asymmetry at rest and during movement, and orofacial muscle tone during the visit. Thirteen children were identified as having no clinical signs of speech motor impairment (NSMI), while 35 children were identified as having evidence of speech motor impairment (SMI). All children with SMI had evidence of dysarthria; therefore, we use the terms SMI and dysarthria interchangeably throughout this paper. Demographic characteristics of the children in the current study are presented in Table 1.

#### 2.1.2. Adult Listeners

Ninety-six adult listeners (2 per child with CP) provided orthographic transcriptions of children’s speech (male = 12, female = 82). Recruitment of listeners involved public postings around the University of Wisconsin-Madison community. Eligibility criteria required the following: age between 18–45 years, be a native speaker of American English, pass a standard pure tone hearing screening, and have no identified cognitive, language, or learning disabilities per self-report. Adult listeners who participated were predominantly undergraduate students. They were compensated either monetarily or with extra credit. Their mean age was 20.89 years (SD = 2.84).

### 2.2. Measures

#### 2.2.1. Transcription Intelligibility Scores

Using a research corpus of 60 multi-word stimuli from the TOCS+ [32] (available via open access in Hustad et al. [9]), speech samples from each participant with CP were collected. Each participant repeated sets of phrases ranging from two to seven words in length (10 of each length), following a pre-recorded auditory model presented on an iPad. The lexical, phonetic, syntactic, and morphological features of the phrases were developmentally appropriate for children and youth. Speech samples were recorded using an Audio-Technica AT4040 condenser studio microphone (Audio-Technica U.S., Inc., Stow, OH, USA), which was positioned 18 inches away from the participant, a Mackie 12,010 VLZ mixer (Mackie Designs Inc., Woodinville, WA, USA), and a Denon DN-500R SD audio recorder (Denon Professional Cumberland, RI, USA) which recorded audio files into .wav files at a 44.1-kHz sampling rate (16-bit quantization). A research assistant monitored the recording quality of each production. Participants were asked to repeat any productions containing co-occurring environmental sounds or speech that overlapped with the examiner.

Research assistants, who had training in acoustic analysis, used Audacity^®^ to segment speech samples into individual utterances and to peak amplitude normalize each utterance. For each participant with CP, segmented utterances were pooled for randomized delivery to adult listeners who transcribed speech for intelligibility measures.

To obtain orthographic transcriptions of intelligibility, adult listeners completed a self-paced task delivered via a customized in-house application in a sound attenuated booth. At the start of each listening session, a research assistant explained to the listener that the goal of the task was to determine how understandable children’s speech productions are to unfamiliar adult listeners. Listeners were informed that an utterance would be played once and were instructed to type what they thought the child said. They were encouraged to take their best guess if they could not fully understand the production. Loudness was calibrated to a peak output level of 75 dB SPL from where the listener was positioned. Presentation of utterances was randomized, and each listener heard all utterances produced by one child. Two different listeners heard each child.

Orthographic transcriptions were automatically scored by the in-house application. Individual words composing each utterance were scored as correct if all phonemes matched the target word from the TOCS+. Homonyms and misspellings were scored as correct when the typed word phonemically matched the spoken version. Transcription intelligibility scores were computed by taking the proportion of words identified correctly for each listener (i.e., correct words divided by total intended words) and averaged across the two listeners per child speaker.

Interrater reliability of transcription intelligibility scores for the two listeners who heard each child was estimated by examining the average difference in intelligibility scores between the two listeners and computing an intraclass correlation coefficient (ICC) using the irr R package (vers. 0.84.1; [34]). The average difference in intelligibility scores between the two listeners for each child speaker was 0.03 proportional point (SD = 0.02). The results of an average-score, consistency-based, one-way random effects model indicated strong agreement among ratings, ICC(2) = 0.997, 95% CI = [0.995, 0.999].

#### 2.2.2. Intelligibility in Context Scale Ratings

Parents of each child completed the ICS at the time of laboratory assessment. Parents were instructed to consider their child’s speech over the past month as they rated their child’s ability to be understood by seven communicative partners that varied in familiarity (i.e., themselves, immediate family, extended family, friends, acquaintances, teachers, and strangers). Each communicative partner comprised a separate item/question on the ICS and was rated using a 5-point ordinal scale, with 1 as the lowest score and 5 as the highest score. To compute the ICS composite score, the average across the seven items was calculated. Thus, each child participant had seven individual ICS item scores (i.e., one for each communicative partner) and an ICS composite score.

### 2.3. Statistical Procedures

In this study, we examined transcription intelligibility scores from adult listeners, ICS composite scores, and ICS individual item scores. Transcription intelligibility scores were continuous proportions between 0 and 1. ICS individual item scores were on an ordinal scale, ranging from 1–5. ICS composite scores were averages of all individual item scores and were continuous between 1 and 5.

To answer the first question examining how well the ICS predicts transcription intelligibility, we used beta regression and estimated marginal means to predict transcription intelligibility scores for the mean ICS composite scores. The beta distribution was used to account for an asymmetric distribution commonly observed with proportions (i.e., intelligibility scores) [35]. Transcription intelligibility proportions were transformed to percentages following statistical analyses for ease of interpretation by multiplying proportions by 100. Proportions are thus reported as percentages throughout the results and discussion sections.

For the second question examining how well individual ICS items predict transcription intelligibility scores, we again used beta regression. We developed a model with all individual ICS items entered simultaneously as predictor variables. This allowed us to examine the total effect of all individual ICS items. To determine the direct effect of each ICS item, we regressed intelligibility onto each ICS item, resulting in seven individual models. To determine partial individual item effects, we used forward selection with likelihood ratio tests to identify statistical significance for added predictors, Akaike information criterion (AIC) differences to compare models, and pseudo $R^{2}$ ($R_{p}^{2}$) differences to identify partial effects.

To address the third question exploring how well ICS composite scores differentiate between children with and without SMI, logistic regression was performed. Odds ratio and probability of having SMI based on ICS composite scores were computed.

Data analysis was executed in R (vers. 4.1.0; [36]) and R studio (vers. 1.4.1717; [37]). Beta regression was completed using betareg R package (vers. 3.1–4; [35]). Marginal means were estimated using the emmeans R package (vers. 1.6.1; [38]). To compute likelihood ratio tests, the lmtest R package (vers. 0.9–38; [39]) was used. AIC differences and R2 differences were determined using the stats R package (vers. 4.1.0; [36]). Logistic regression, odds ratio, and probability were computed using the rms R package (vers. 6.2–0; [40]).

## 3. Results

**Figure 1:**
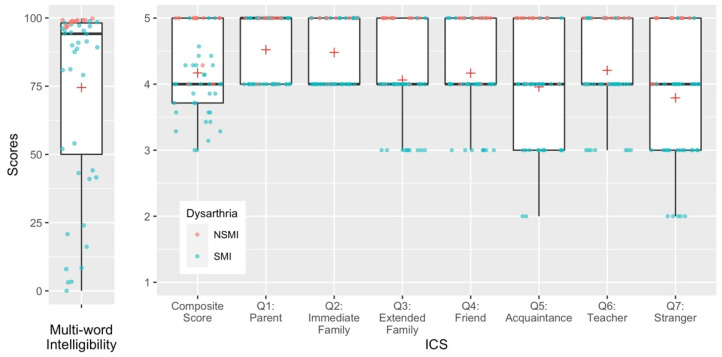
Distribution of transcription intelligibility and ICS scores with observed data points from participants. Group mean scores are marked with a red cross. Participant data points are categorized into no clinical signs of speech motor impairment (NSMI) and evidence of speech motor impairment (SMI).

Descriptive results for children’s transcription intelligibility and ICS scores are presented in Figure 1. Note that ratings for each ICS item ranged from 2 to 5. The rating of 2 was rarely used and the rating of 1 was never used, even for children with intelligibility scores near zero.

### 3.1. Research Question 1: How Well Do ICS Composite Scores Predict Transcription Intelligibility Scores?

Using a beta regression model of transcription intelligibility scores as a function of ICS composite scores, the results of a likelihood ratio test showed a statistically significant relationship, χ^2^ (2, N = 48) = 17.11, *p* < 0.001. That is, change in transcription intelligibility scores was associated with change in ICS composite scores. The odds ratio was 3.5, indicating a moderate effect size [41]. The pseudo $R^{2}$ ($R_{p}^{2}$) value indicated that 40.17% of the variance in transcription intelligibility scores was explained by ICS composite scores.

**Figure 2:**
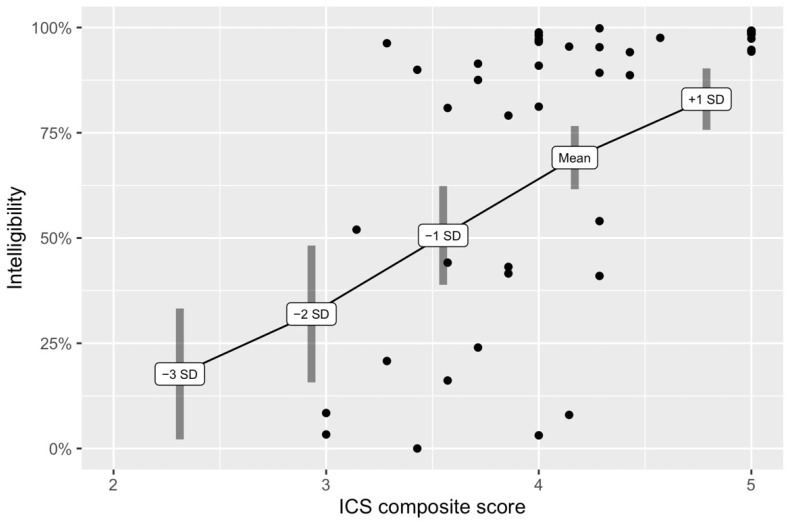
Predicted transcription intelligibility scores based on marginal ICS composite score mean with observed data points from participants.

The estimated marginal mean of ICS composite scores was 4.17. The predicted transcription intelligibility score for the mean ICS composite score was 69.10%, 95% CI [61.59%, 76.61%] (Table 2). One standard deviation above (+1 SD) the mean ICS composite score estimated a 13.89% increase in transcription intelligibility score (Figure 2). One standard deviation below (−1 SD) the mean ICS composite score estimated an 18.49% decrease in transcription intelligibility score. Two standard deviations below (−2 SD) the mean ICS composite score estimated a 37.14% decrease in transcription intelligibility score. Three standard deviations below (−3 SD) the mean ICS composite score estimated a 51.39% decrease in transcription intelligibility score. Note that the confidence intervals for the predicted transcription intelligibility values at −2 SD and −3 SD were wider due to fewer available data points in that range.

Figure 2 shows individual participant data plotted with predicted transcription intelligibility scores based on marginal ICS composite score means. Results indicate that individuals with an ICS composite score between 3 and 4 varied widely in terms of transcription intelligibility scores. The majority of the participants who received an ICS composite score above four (78.26%, *n* = 18 of 23) had high speech intelligibility (>90%) [19].

### 3.2. Research Question 2: Do Individual Questions from the ICS Differentially Predict Transcription Intelligibility Scores?

Regression of transcription intelligibility scores onto all ICS item individual scores revealed that 63.81% of the variance in transcription intelligibility scores was explained ($R_{p}^{2}$ = 0.6381), which was higher than the previous model that used only the ICS composite scores (Research Question 1). The difference in AIC for the regression model including ICS composite scores (Research Question 1) and the regression model with all seven ICS items (Research Question 2) was 14.88, indicating that the regression model with all seven ICS items was a better model for predicting intelligibility scores.

Table 3 presents the AIC and $R_{p}^{2}$ values of beta regression models between transcription intelligibility and each ICS item. Question 7, which asked “Do strangers understand your child?” (Q7: Stranger) had the highest $R_{p}^{2}\;$value, indicating the largest direct effect, and lowest AIC, indicating the best fit compared to the other models.

The beta regression model for Q7: Stranger was compared to all potential two-predictor models (i.e., Q7: Stranger with each one of the remaining ICS items). Table 4 summarizes the results of likelihood ratio tests, AIC differences, and $R_{p}^{2}\;$differences. Based on the AIC differences, the two-predictor model with Q6: Teacher (i.e., Do your child’s teachers understand your child?) as an added predictor was identified as the best fitting model compared to other potential two-predictor models. However, the $R_{p}^{2}\;$difference indicated only a 2% increase in the explained variance compared to the model having only Q7: Stranger as a predictor. The results of the likelihood ratio test and computation of AIC difference indicated no statistically significant difference between the models (i.e., Q7: Stranger as the only predictor compared to Q7: Stranger + Q6: Teacher as predictors), thus the forward selection process was discontinued because adding another predictor would have minimal effects on transcription intelligibility.

### 3.3. Research Question 3: How Well Do ICS Composite Scores Differentiate between Children with and without Speech Motor Impairment?

**Figure 3:**
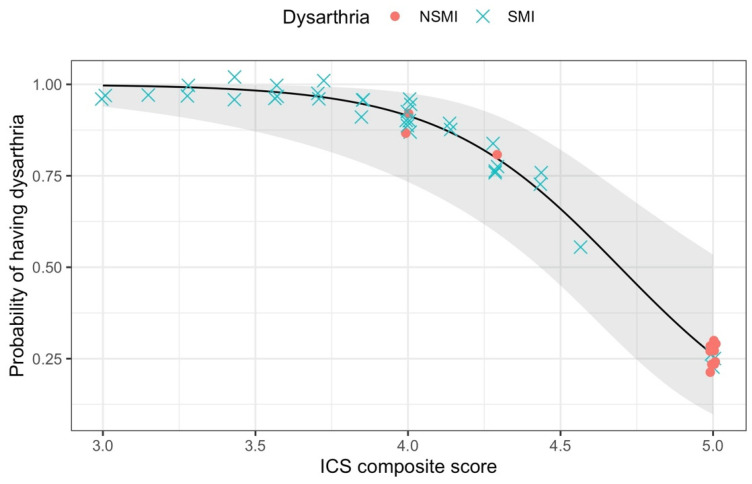
Probability of having SMI given specific ICS composite scores based on the logistic model of presence of SMI as a function of ICS composite score with actual data points from participants. NSMI = No clinical speech motor impairment. SMI = Speech motor impairment.

ICS composite scores of participants with NSMI ranged from 4 to 5 (M = 4.79, SD = 0.40). ICS composite scores of participants with SMI ranged from 3 to 5 (M = 3.94, SD = 0.52). Results of logistic regression modeling for the presence of SMI as a function of ICS composite scores showed strong evidence that ICS composite scores could indicate the presence or absence of SMI, χ^2^ (1, N = 48) = 22.16, *p* < 0.001. A shift in ICS composite score from 4.79 to 3.94 (i.e., using the group means) resulted to an odds ratio of 18.07 [95% CI (3.71, 87.97)], indicating that children who received an ICS composite score of 3.94 had 18.07 times higher likelihood of having SMI compared to children who received an ICS composite score of 4.79. Figure 3 shows that as ICS composite scores increased, the probability of having SMI decreased.

Individuals with an ICS score of 4 had a 91.41% probability of having SMI, with 95% CI (73.32, 97.63). Logistic regression results suggested that children with CP whose ICS composite score was 4 or lower had higher probabilities of having SMI.

Individuals with an ICS composite score of 5 had a 26.10% probability of having SMI [95% CI (9.80, 53.45)]. Note that there were fewer participants with NSMI than there were with SMI. There were also several participants with SMI who had ICS composite scores of 5. Both of these likely reduced the precision of the prediction, thus resulting in wide confidence intervals.

## 4. Discussion

This study sought to quantify the relationship of ICS scores to transcription intelligibility scores for the speech of children with CP. Specifically, we examined how well ICS composite scores predict transcription intelligibility scores, whether individual ICS items differentially predict transcription intelligibility scores, and how well ICS composite scores differentiate between children with and without SMI. To address these questions, we examined intelligibility scores based on orthographic transcription by naïve listeners and parent ratings from the ICS for 48 children with CP who were approximately 13 years of age. The main findings from this study were: (1) ICS composite scores predicted transcription intelligibility scores with a moderate effect size; (2) individual ICS items differentially predicted transcription intelligibility scores, with Q7:Stranger (i.e., Do strangers understand your child?) being the best predictor; and (3) ICS composite scores below 4 indicated higher likelihood of having SMI, while ICS composite scores above 4 indicated lower likelihood of having SMI. Each main finding is discussed in detail below.

### 4.1. ICS Composite Scores Predicted Transcription Intelligibility Scores with a Moderate Effect Size

In this study, we found that ICS composite scores made a significant contribution to the prediction of transcription intelligibility scores; however, more than half of the variance in transcription intelligibility scores was not explained by ICS composite scores. Findings were consistent with our hypothesis that ICS composite scores would be a moderately strong predictor of transcription intelligibility scores. Individual data shown in Figure 2 suggest that this moderate effect size may be due to wide variability among children whose ICS composite scores were between 3 and 4. In some cases, parent ratings on the ICS were relatively high on the same children for whom transcription intelligibility scores from unfamiliar listeners were relatively low. Notably, transcription intelligibility scores for children with ICS composite scores between 3 and 4 were as high as 99% and as low as 0%. Composite ICS scores did not fall below 3 in the dataset, even for children with transcription intelligibility scores at or near 0%. Lagerberg and associates also reported a wide range of transcription intelligibility scores for ICS composite scores of 4 and below [30], which supports the notion that internal calibrations of parents regarding their child’s intelligibility as rated by the ICS may vary [19].

One potential conclusion from the discrepant findings between transcription intelligibility scores and ICS composite scores for some children is that ICS composite scores and transcription intelligibility scores may not be capturing the same thing. For example, rating scales, such as the ICS, seek to characterize general and cumulative exposure to a child’s speech, giving a gross impression of intelligibility in communicative contexts [22,42]. Transcription intelligibility scores are based on an isolated and focused exposure with a child’s speech, capturing objective details from specific speech samples in the absence of communicative context [7,14]. Our findings are similar to those of Sakash and colleagues [19] and Lagerberg and collaborators [30], who suggested that parent ratings of intelligibility and transcription intelligibility scores were correlated but were not equivalent measures. They advocated for the use of these two measures together when assessing speech intelligibility.

In contrast, Hossienabad and associates [31] recommended the use of the ICS as a functional measure of intelligibility for children with VPI, as they observed that the ICS strongly correlated with transcription intelligibility scores, clinician intelligibility ratings, and articulation performance. A potential explanation for the difference in results from the present study relates to the speech manifestations of dysarthria versus VPI. Dysarthria can lead to difficulty with all or several subsystems of speech, including respiration, phonation, resonation, articulation, and prosody [43,44]. VPI predominantly affects resonation and articulation [31,45], which may lead to more predictable speech production features than dysarthria. As individuals with dysarthria may have varied underlying subsystem impairments that lead to reduced intelligibility, using ICS ratings in complement with transcription intelligibility scores may permit a more comprehensive characterization of functional speech abilities than either measure alone.

### 4.2. Individual ICS Items Differentially Predicted Transcription Intelligibility Scores

The ICS was designed to capture a child’s intelligibility across different communication partners. Our results aligned with our hypothesis that individual ICS items would have differential effects on prediction of transcription intelligibility scores. Parents seemed to be aware that their child’s intelligibility varied with different communication partners. However, their ratings did not tend to vary widely across partners. Specifically, few parents used more than two levels of the Likert scale across all partners, and they rarely used the lowest ratings even for less familiar communication partners. Interestingly, however, individual ICS item scores considered simultaneously as a group explained more variance in intelligibility scores than ICS composite scores. This finding highlights the importance of evaluating individual environmental and contextual factors when measuring speech intelligibility. Considering all individual item scores, together with ICS composite scores, during clinical assessment may provide useful information for preparing a more comprehensive intervention plan [20].

When we examined which ICS items or combinations of items were the best predictors of transcription intelligibility scores, we found that only item Q7:Stranger was a meaningful predictor. In fact, scores on this question alone had a larger effect size for the prediction of transcription intelligibility scores than the ICS composite score. One explanation can be observed in Figure 1. Raw data on ICS ratings for Q7: Stranger suggest that parents used the greatest range of ratings on this question, reflecting closer calibration with transcription intelligibility scores from unfamiliar listeners. This finding suggests that parents may have reasonably good insight into difficulties that unfamiliar listeners might have. Other literature [21] has demonstrated that familiarity seems to have an impact on ICS ratings, with parents rating themselves the highest (see Figure 1), and strangers the lowest. Studies examining the impact of listener familiarity on transcription intelligibility scores are needed to begin to understand how parent perceptions of intelligibility with different partners map onto their actual performance when presented with speech.

It was also interesting that item Q1:Parent (i.e., Do you understand your child?) was the weakest predictor of transcription intelligibility scores, accounting for only 9% of the variance. This finding highlights the discrepancy between parent perceptions of themselves and the performance of unfamiliar listeners. Again, there may be truth to this finding in that parents are very likely “better” listeners than unfamiliar partners, with higher transcription intelligibility scores for their own children than naïve listeners. However, further research is needed to investigate this question, which in turn would advance our understanding of the extent to which rating scale findings for intelligibility reflect transcription results for intelligibility as measured by the same listeners.

### 4.3. ICS Composite Scores below 4 Indicated Higher Likelihood of Having Dysarthria

A key finding from this study was that children with ICS composite scores of 4 or lower had a probability greater than 90%, on average, of having SMI. This finding was similar to observations from previous studies that ICS composite scores can differentiate children with SSD from typically developing children [20]. However, children with an ICS composite score of 5 also had a probability of about 25% of having SMI. As noted previously, the precision of predicting SMI status was lower for ICS composite scores above 4 (i.e., wider confidence intervals for ICS composite scores above 4 in Figure 2). Examination of individual child data may explain this observation. Transcription intelligibility scores of three children with NSMI ranged from 96.60% to 99.81%, and they received an ICS composite score that ranged from 4 to 4.28. However, three other children with SMI received ICS composite scores of 5. Notably, they had mild dysarthria related to phonation and tongue strength based on oral-motor assessment. Speech distortions were noted but mildly affected their intelligibility (i.e., their transcription intelligibility rating ranged from 94.27% to 98.49%). This suggests that severity of dysarthria (often measured by intelligibility) influenced how well the ICS predicted SMI status. Inferences from individual child data indicate that ICS composite scores correctly identified children with SMI with notable intelligibility reduction; however, ICS composite scores were not sensitive at identifying children with SMI who had high intelligibility. The observation that ICS composite scores separated children with dysarthria who had high intelligibility from those who had reduced intelligibility indicates that ICS composite scores were more sensitive to differences in intelligibility than the presence of dysarthria. As the intended purpose of the ICS is to measure intelligibility differences, this was a logical outcome.

A key conclusion is that the clinical utility of the ICS as a screening tool for the presence of dysarthria is mediated by the severity level of dysarthria. Future studies should consider severity as a variable in the prediction of ICS scores to further examine the potential clinical utility of the ICS as a screening tool for the presence of dysarthria, particularly in young children. Findings from this study suggested that composite scores were more accurate at predicting the presence of dysarthria among children with reduced intelligibility, but did not readily differentiate children with dysarthria who had higher intelligibility from those who did not have dysarthria. Another potential follow-up study could explore the potential clinical utility of the ICS item Q7: Stranger for predicting dysarthria.

## 5. Limitations and Future Directions

The current study included a relatively small sample of children with CP. Furthermore, there were fewer participants with no clinical SMI, which may have contributed to the wide confidence intervals when predicting the presence of dysarthria among children who received an ICS composite score above 4. Further investigation with a larger sample of children with CP is recommended to replicate the current findings.

Our sample only included children who were approximately 13 years of age, representing school aged children. Future research that includes younger age groups is important for describing the utility of the ICS among children with CP across different developmental stages.

Available research exploring the relationship between ICS ratings and transcription intelligibility scores has focused on children with speech disorders. The clinical utility of the ICS as a screening tool can be furthered by investigating the relationship between ICS ratings and transcription intelligibility scores among typically developing children. Findings from such research may provide useful benchmarks for assessment and for monitoring progress. Thus, future research should include large scale normative studies of the ICS with typical children across a wide range of ages.

## Figures and Tables

**Table 1:** Demographic characteristics of children with cerebral palsy.

	NSMI (*n* = 13)	SMI (*n* = 35)	Total (*n* = 48)
Male:female ratio	9:4	19:16	28:20
Age in years: Mean (SD)	13.01 (1.92)	12.74 (1.97)	12.82 (1.94)
Cerebral palsy type			
Spastic	13	26	39
Diplegia	4	6	10
Hemiplegia (left)	6	5	11
Hemiplegia (right)	3	9	12
Triplegia	0	1	1
Quadriplegia	0	4	4
Not reported	0	1	1
Ataxic	0	4	4
Hypotonic	0	1	1
Mixed	0	1	1
Unknown	0	3	3
Dyskinetic	0	0	0
GMFCS			
I	12	12	24
II	1	15	16
III	0	4	4
IV	0	2	2
V	0	2	2
MACS			
I	6	10	16
II	7	16	23
III	0	8	8
IV	0	1	1
V	0	0	0

SD = Standard Deviation; NSMI = No speech motor impairment; SMI = Speech motor impairment; GMFCS = Gross Motor Function Classification System; MACS = Manual Ability Classification System.

**Table 2:** Predicted transcription intelligibility scores based on marginal ICS composite score mean.

				95% Confidence Interval	
	ICS Score	Intelligibility Score	Standard Error	Lower Boundary	Upper Boundary
−3 SD	2.31	17.71%	0.08	2.16%	33.26%
−2 SD	2.93	31.96%	0.08	15.70%	48.21%
−1 SD	3.55	50.61%	0.06	38.87%	62.35%
Mean	4.17	69.10%	0.04	61.59%	76.61%
+1 SD	4.79	82.99%	0.04	75.69%	90.30%

Note: ICS = Intelligibility in Context Scale.

**Table 3:** AIC and pseudo R-squared value of beta regression models.

ICS Items	AIC	$R_{p}^{2}$
Q7: Stranger	−84.04	0.53
Q5: Acquaintance	−75.72	0.43
Q4: Friend	−74.63	0.41
Q3: Extended Family	−74.36	0.41
Q6: Teacher	−61.90	0.16
Q2: Immediate Family	−60.78	0.13
Q1: Parent	−59.34	0.09

Note: AIC = Akaike information criterion; Q = Question; $R_{p}^{2}$ = pseudo R-squared.

**Table 4:** Likelihood ratio test, AIC differences, and pseudo R-squared differences when comparing the beta regression model of Q7: Stranger as a predictor with all potential two-predictor models.

ICS Items	Chi Square	*p*-Value	AIC Difference	$R_{p}^{2}$ Difference
Q7 (Stranger) + Q6 (Teacher)	2.72	0.10	0.72	0.02
Q7 (Stranger) + Q1 (Parent)	1.07	0.30	−0.93	0.01
Q7 (Stranger) + Q2 (Immediate Family)	0.94	0.33	−1.06	0.01
Q7 (Stranger) + Q4 (Extended Family	0.64	0.43	−1.36	0.01
Q7 (Stranger) + Q5 (Friend)	0.09	0.77	−1.91	0.00
Q7 (Stranger) + Q3 (Extended Family)	0.00	0.95	−2.00	0.00

Note: AIC = Akaike information criterion; Q = Question; $R_{p}^{2}$ = pseudo R-squared.

## Data Availability

The data presented in this study are available upon request. The data are not publicly available due to human subjects privacy restrictions.
